# Dynamics of progressive degeneration of major spinal pathways following spinal cord injury: A longitudinal study

**DOI:** 10.1016/j.nicl.2023.103339

**Published:** 2023-02-01

**Authors:** Simon Schading, Gergely David, Tim Max Emmenegger, Cristian Achim, Alan Thompson, Nikolaus Weiskopf, Armin Curt, Patrick Freund

**Affiliations:** a Spinal Cord Injury Center, Balgrist University Hospital, University of Zurich, Zurich, Switzerland; b Department of Brain Repair and Rehabilitation, UCL Queen Square Institute of Neurology, University College London, London, UK; c Department of Neurophysics, Max Planck Institute for Human Cognitive and Brain Sciences, Leipzig, Germany; d Felix Bloch Institute for Solid State Physics, Faculty of Physics and Earth Sciences, Leipzig University, Leipzig, Germany; e Wellcome Trust Centre for Neuroimaging, UCL Queen Square Institute of Neurology, University College London, London, UK

**Keywords:** AIS, American Spinal Injury Association Impairment Scale, APW, anterior-posterior width, CST, corticospinal tract, DC, dorsal columns, ISNCSCI, International Standards for Neurological Classification of Spinal Cord Injury, LRW, left–right width, MPM, multi-parameter mapping, MT, magnetization transfer, MTsat, magnetization transfer saturation, PD, proton density, p.u., percentage unit, SCI, spinal cord injury, Spinal cord injury, Quantitative MRI, Neurodegeneration, Biomarker, Demyelination

## Abstract

**Background:**

Following spinal cord injury (SCI), disease processes spread gradually along the spinal cord forming a spatial gradient with most pronounced changes located at the lesion site. However, the dynamics of this gradient in SCI patients is not established.

**Objective:**

This study tracks the spatiotemporal dynamics of remote anterograde and retrograde spinal tract degeneration in the upper cervical cord following SCI over two years utilizing quantitative MRI.

**Methods:**

Twenty-three acute SCI patients (11 paraplegics, 12 tetraplegics) and 21 healthy controls were scanned with a T1-weighted sequence for volumetry and a FLASH sequence for myelin-sensitive magnetization transfer saturation (MTsat) of the upper cervical cord. We estimated myelin content from MTsat maps within the corticospinal tracts (CST) and dorsal columns (DC) and measured spinal cord atrophy by means of left–right width (LRW) and anterior-posterior width (APW) on the T1-weighted images across cervical levels C1-C3. MTsat in the CST and LRW were considered proxies for retrograde degeneration, while MTsat in the DC and APW provided evidence for anterograde degeneration, respectively. Using regression models, we compared the temporal and spatial trajectories of these MRI readouts between tetraplegics, paraplegics, and controls over a 2-year period and assessed their associations with clinical improvement.

**Results:**

Linear rates and absolute differences in myelin-sensitive MTsat indicated retrograde and anterograde neurodegeneration in the CST and DC, respectively. Changes in MTsat within the CST and in LRW progressively developed over time forming a gradient towards lower cervical levels by 2 years after injury, especially in tetraplegics (change per cervical level in MTsat: −0.247 p.u./level, p = 0.034; in LRW: −0.323 mm/level, p = 0.024). MTsat within the DC was already decreased at cervical levels C1-C3 at baseline (1.5 months after injury) in both tetra- and paraplegics, while linear decreases in APW over time were similar across C1-C3, preserving the spatial gradient. The relative improvement in light touch score was associated with MTsat within the DC at baseline (r_s_ = 0.575, p = 0.014).

**Conclusion:**

Rostral and remote to the injury, the CST and DC show ongoing structural changes, indicative of myelin reductions and atrophy within 2 years after SCI. While anterograde degeneration in the DC was already detectable uniformly at C1-C3 early following SCI, retrograde degeneration in the CST developed over time revealing specific spatial and temporal neurodegenerative gradients. Disentangling and quantifying such dynamic pathological processes may provide biomarkers for regenerative and remyelinating therapies along entire spinal pathways.

## Highlights

• Retro- and anterograde degeneration show different spatiotemporal dynamics post-SCI.
• Retrograde degeneration gradually develops and forms a spatial gradient over time.
• Anterograde degeneration is detectable early post-SCI without a spatial gradient.
• Tract-specific microstructural changes are predictive of functional improvement.

## 1 Introduction

Traumatic spinal cord injury (SCI) is a devastating event with an enormous impact on patients' quality of life, due to sensorimotor and autonomous deficits resulting from disrupted ascending and descending fibre pathways. Degeneration and demyelination of spinal pathways are major limitations to functional recovery and currently there is no intervention which can prevent this occurring (Curt et al., 2008). After SCI, the primary injury triggers a cascade of pathophysiological processes at the focal injury site (Ahuja et al., 2017) with subsequent secondary disease processes (i.e. anterograde, retrograde, and trans-synaptic degeneration) (David et al., 2021, David et al., 2019, David et al., 2022, Freund et al., 2019, Freund et al., 2013b, Grabher et al., 2015, Lundell et al., 2011, Seif et al., 2018a, Seif et al., 2020, Ziegler et al., 2018). Histopathological evidence illustrates that at the earliest stage post-SCI axonal swelling and myelin thinning can be detected at the lesion border (Brennan et al., 2013, Bresnahan, 1978, Bresnahan et al., 1976, Buss et al., 2005, Grumbles and Thomas, 2017). Over time these processes spread gradually along the spinal cord (i.e. neurodegenerative gradient) with the most pronounced changes located at the lesion site (Bresnahan, 1978, Bresnahan et al., 1976, Grumbles and Thomas, 2017). However, the dynamics of this gradient in sub-acute SCI patients is not established. Importantly, evidence from histopathological studies suggests that anterograde and retrograde neurodegeneration are two distinct entities each with its own temporal and spatial properties (Bresnahan, 1978, Daniel and Strich, 1969, Hill, 2017, Kalil and Schneider, 1975). Insights into such dynamic processes, in particular by using non-invasive MRI provide greater understanding of the pathophysiological processes following SCI which may ultimately help to improve prognostication (Schading et al., 2021).

Quantitative MRI, as implemented in the multi-parameter mapping (MPM) protocol, allows the assessment of changes on a microstructural level (Weiskopf et al., 2013). In particular, magnetization transfer saturation (MTsat), which represents the fraction of saturated free water spins following an off-resonance MT pulse (Helms et al., 2008), has been shown to be closely associated with myelin content (Georgiadis et al., 2021, Schmierer et al., 2004, Weiskopf et al., 2021). Here, we analyzed myelin-sensitive MTsat maps and structural T1-weighted images to track neurodegenerative changes in micro- and macrostructure across the upper cervical cord over two years following SCI and evaluated the dependency of neurodegeneration on the distance to the lesion. In contrast to a previous report (Azzarito et al., 2020), we investigate the neurodegenerative gradient not only spatially (across cervical levels) but also longitudinally.

Moreover, anterograde and retrograde neurodegeneration show distinct pathophysiological changes (Bresnahan, 1978). Thus, we investigated them separately by focusing on changes in the corticospinal tracts (CST) encompassing descending motor fibers and the dorsal columns (DC) encompassing ascending sensory fibers, respectively. As the imaging region was above the injury site, changes of the CST served as proxies for retrograde degeneration, while changes in the DC provided evidence for anterograde degeneration.

Based on existing evidence (Azzarito et al., 2020, Bresnahan, 1978, Grumbles and Thomas, 2017, Hill, 2017, Kalil and Schneider, 1975), we hypothesized that (i) the rate of neurodegenerative changes is more pronounced closer to the lesion, which was below C3 (i.e. C3 > C2 > C1); (ii) the differences in atrophy rates between cervical levels lead to the development of a neurodegenerative gradient; and (iii) anterograde and retrograde axonal degeneration exhibit different spatiotemporal dynamics, which relate to the underlying pathophysiological mechanisms.

## 2 Materials and Methods

### 2.1 Participants and study design

23 patients with acute traumatic and non-traumatic SCI (11 paraplegics, 12 tetraplegics), admitted consecutively to the rehabilitation program at the Balgrist University Hospital (Zurich, Switzerland) between September 2010 and August 2014 (Table 1), and 21 healthy controls participated in this longitudinal study (data or parts of data already reported) (Azzarito et al., 2020, Freund et al., 2013b, Grabher et al., 2015, Seif et al., 2018a, Seif et al., 2018b, Ziegler et al., 2018). Eligible patients were older than 18 years and showed no signs of concomitant head or brain lesions. Further exclusion criteria were pre-existing neurological or mental disorders, medical disorders leading to functional impairment, or contraindications to MRI. SCI patients were scanned at baseline (n = 22), 2-month (n = 20), 6-month (n = 22), 1-year (n = 21), and 2-year (n = 17) follow-up. In patients, the mean (±standard deviation [SD]) interval from the date of injury to the first (baseline) scan was 1.5 (±0.7) months, to the second 3.6 (±1.4) months, to the third 7.3 (±2.0) months, to the fourth 13.7 (±3.3) months, and to the fifth 28.1 (±4.3) months. Healthy controls followed the same imaging schedule. Additionally, SCI patients underwent a comprehensive clinical examination protocol according to the International Standards for Neurological Classification of Spinal Cord Injury (ISNCSCI) for the assessment of motor, light touch and pin-prick score (Kirshblum et al., 2011).

**Table 1:** Patient information.

	ID	Age at injury [years]	sex	AIS	initial neurological level of injury	Level of hyperintensive signal change	injury mechanism
pSCI	1	46	F	D	T8	T6-T12	Prolapsed disc T8/9 with spinal cord ischemia
	2	67	F	B	T9	T6-T12	AV-fistula T9-T11 with myelopathy
	3	74	M	D	T10	-a	Dislocation fracture T12/L1
	4	77	F	D	T10	T9-conus medullaris	Aortic embolism with spinal cord ischemia
	5	43	M	D	T11	none	Massively prolapsed disc L3/4
	6	52	M	D	T9	-a	Flexion-distraction T9/10
	7	29	M	A	T11	-a	Flexion-distraction T12/L1 & burst fracture L1
	8	71	M	C	T10	T10-12	Spinal cord ischemia
	9	71	F	D	T3	T6-T8	Discogenic spinal cord compression T3-T9
	10	72	F	A	T11	T10-T12	Spinal epidural hematoma
	11	31	M	C	T2	T2-T7	Dislocation fracture T4/5

tSCI	1	18	M	A	C5	C5-C7	Burst fracture C6
	2	22	M	B	C7	C6/7	Dislocation fracture C6/7
	3	41	M	A	C5	C6/7	Anterolisthesis of C6 relative to C7
	4	70	M	B	C7	C7	Hyperflexion C6/7
	5	30	M	B	C7	C6/7	Dislocation fracture C6/7
	6	47	M	D	C4	C6/7	Dislocation fracture C6/7
	7	51	M	B	C6	C6/7	Dislocation fracture C6/7
	8	67	M	D	C3	C3/4	Spinal cord compression C3/4 & acute contusion
	9	32	M	A	C5	C6/7	Flexion-distraction C5-C7
	10	53	F	C	C4	C4/5	Spinal cord compression C4/5
	11	30	M	A	C4	C5/6	Dislocation fracture C5/6 & burst fracture C6
	12	28	M	A	C4	C4/5	Dislocation fracture C4/5

*AIS* American Spinal Injury Association Impairment Scale, *pSCI* paraplegic spinal cord injury patients, *tSCI* tetraplegic spinal cord injury patients.

a Spinal cord cannot be assessed at the level of injury due to artifacts caused by metal implants.

All participants provided written informed consent before participation in the study, which was approved by the local Ethics Committee of Zurich (EK-2010–0271).

### 2.2 Image acquisition

MRI scanning was performed on clinical 3 T Siemens Verio and 3 T Siemens Skyra scanners (Erlangen, Germany), using the body transmit radio-frequency (RF) coil and a 16-channel receive head/neck RF coil. A T1-weighted 3D Magnetization Prepared Rapid Acquisition Gradient-Echo (MPRAGE) sequence covering the brain and cervical levels C1-C5 was acquired with the following parameters: 176 slices, in-plane field of view (FOV): 224x256 mm^2^, 1 mm^3^ isotropic resolution, repetition time (TR): 2420 ms, echo time (TE): 4.18 ms, flip angle: 9°, inversion time (TI): 960 ms, readout bandwidth: 150 Hz/pixel, total scan time: 9.04 min. The multi-parameter mapping (MPM) protocol (Weiskopf et al., 2013), consisting of three 3D multi-echo fast low-angle shot (FLASH) gradient-echo sequences with different contrast weightings and parameters (T1-weighted, proton-density (PD)-weighted, and magnetization-transfer (MT)-weighted), was implemented based on product sequences available on the respective MRI scanner (Leutritz et al., 2020) and was acquired with identical slice prescription covering the brain and cervical levels C1-C5. All three sequences were acquired with 176 slices, FOV: 240x256 mm^2^, 1 mm^3^ isotropic resolution, parallel imaging with GRAPPA 2x, and readout bandwidth: 480 Hz/pixel. TR and flip angle were 25 ms/23° (T1-weighted), 25 ms/4° (PD-weighted), and 37 ms/9° (MT-weighted). Six (MT-weighted) and eight (PD- and T1-weighted) echoes were acquired (echo spacing: 2.46 ms, TE of first echo: 2.46 ms). MT weighting was achieved by applying an off-resonance RF pulse (Gaussian shape, pulse length: 10 ms, MT pulse flip angle: 500°, off-resonance frequency: 1.2 kHz, bandwidth: 192 Hz) prior to non-selective excitation. The total scan time for MPM was 23 min.

### 2.3 MRI data processing

**Fig. 1:**
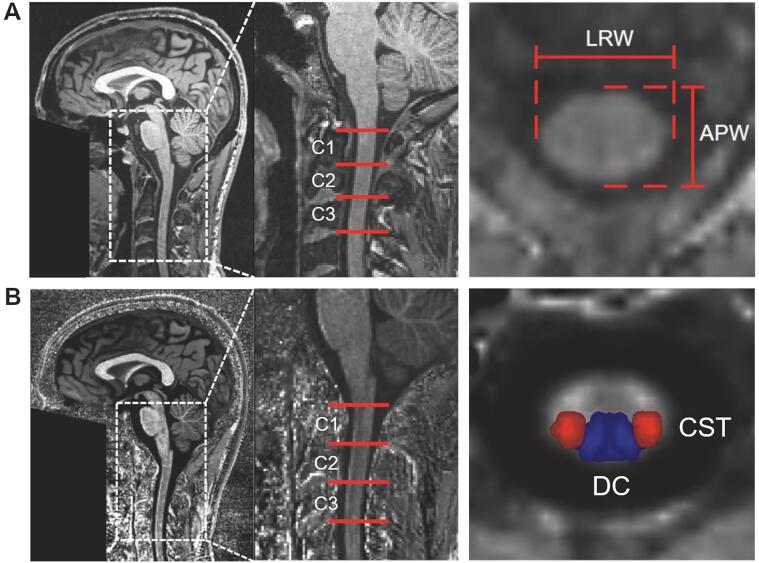
**Illustration of morphometric parameters and white matter tracts.** (A) Sagittal section of a T1-weighted image with indication of cervical levels C1-C3 and illustration of macrostructural parameters left–right width (LRW) and anterior-posterior width (APW) on a transversal section of the T1-weighted image. (B) Sagittal section of a MTsat map with indication of cervical levels C1-C3 and overlay of spinal cord white matter tracts (corticospinal tract [CST], dorsal columns [DC]) on a transversal section of the MTsat map for the extraction of tract-specific microstructural parameters. *APW* anterior-posterior width, *CST* corticospinal tracts, *DC* dorsal columns, *LRW* left–right width, *MTsat* magnetization transfer saturation.

Maps of MTsat were calculated from the T1-weighted, PD-weighted and MT-weighted scans using the “Create hMRI maps” module of the SPM12-based hMRI toolbox (v0.2.0) (Tabelow et al., 2019), applying UNICORT correction for RF transmit field B1+ inhomogeneities (Weiskopf et al., 2011) and no correction for imperfect spoiling. T1-weighted MPRAGE images and MTsat maps were processed with the Spinal Cord Toolbox (De Leener et al., 2017) as follows: MPRAGE images and MTsat maps were segmented for spinal cord using the deep learning-based *sct_deepseg_sc* algorithm (Gros et al., 2019) and the resulting binary masks were corrected manually if necessary. Both sets of images were co-registered with the MNI-Poly-AMU template (De Leener et al., 2018) after semiautomatic identification of vertebral levels C1-C3. Subsequently, the white matter atlas (Lévy et al., 2015) (comprising anatomical masks of the CST and DC) was warped into each subject's space. Morphometric parameters including anterior-posterior width (APW) and left–right width (LRW) were extracted from the MPRAGE images at vertebral levels C1-C3 (Fig. 1A). Using the white matter atlas of the MNI-Poly-AMU template (De Leener et al., 2018), MTsat values were averaged across voxels within the CST and DC at the same vertebral levels (Fig. 1B). As the imaging region was above the injury site, MTsat in the CST and LRW were considered proxies for retrograde degeneration, while MTsat in the DC and APW provided evidence for anterograde degeneration, respectively (Grabher et al., 2015, Jutzeler et al., 2016, Lundell et al., 2011, Ziegler et al., 2018).

### 2.4 Statistical analysis

All statistical analyses were performed in RStudio (Version 4.0.5). Differences between tetraplegics, paraplegics, and controls were tested using Kruskal-Wallis test and post-hoc Dunn-Bonferroni test for age, Fisher's exact test for the American Spinal Injury Association Impairment Scale (AIS) and sex, and Wilcoxon rank-sum test for scan time points with a significance threshold of p = 0.05.

Images were carefully inspected at each vertebral level and the respective vertebral levels were excluded in case of extensive susceptibility and/or motion artifacts. Therefore, the final sample size was 83% (C1), 83% (*C*2), 79% (C3) of the total sample size for MPRAGE, and 74% (C1), 74% (*C*2), 64% (C3) for MTsat.

To investigate spatiotemporal changes in MRI readouts, a linear mixed effect model was created for each readout, with fixed effects being the vertebral level (3 levels: C1-C3), time since injury (5 time points), as well as their interaction with group (3 groups: controls, tetraplegics, paraplegics). For morphological readouts (APW, LRW), a quadratic term of time since injury was also added as fixed effect to model the deceleration of atrophy. Random effects were the individual intercept and slope associated with time since injury and vertebral level, and the quadratic term of time for morphological readouts. Furthermore, the interaction of age with time since injury was added to account for aging-related changes (Papinutto et al., 2020). Since the MRI scanner was upgraded over the course of the study, the models were additionally corrected for the scanner type. Post-hoc pairwise comparison with Tukey's correction (p < 0.05) was used to compare the linear and quadratic effects between the groups, the spatial trajectories across cervical levels C1-C3 at five time points (baseline, 2-month, 6-month, 1-year, and 2-year follow-up), as well as the average on each cervical level (C1-C3) at the beginning and the end of the observation period (baseline and 2-year follow-up).

To assess associations between microstructural MRI readouts and clinical measures, a linear mixed effects model was created for each clinical measure (total motor score, light touch score) with the fixed effect being the logarithmic time since injury to accommodate nonlinear recovery (Curt et al., 2008) and its interaction with group (2 groups: paraplegics, tetraplegics), and the random effects being the individual intercept and the logarithmic time since injury. Moreover, these models were adjusted for potentially confounding effects of age. The relative clinical improvement was calculated as follows:

$$\text{Relative}\;\text{Improvement}\;\left[\%\right]=\;\frac{\;{score}_{\mathrm{follow}-up}-{score}_{\mathrm{baseline}}}{{\mathrm{score}}_{\text{max}}-{score}_{\mathrm{baseline}}\;}\cdot 100$$

where ${\mathrm{score}}_{\mathrm{baseline}}$ and ${\mathrm{score}}_{\mathrm{follow}-up}$ denote the baseline and follow-up clinical scores, respectively, and ${\mathrm{score}}_{\max}$ denotes the maximum reachable score. Thereby, relative improvement represents the percentage of actually achieved improvement compared to the total possible improvement. Spearman's rank correlation was calculated between the estimated MTsat within the DC and the CST across C1-C3 at baseline and the relative improvement in light touch score and total motor score between baseline and 2-year follow-up, respectively.

## 3 Results

### 3.1 Patients' characteristics and clinical outcomes

Demographic and clinical information of the SCI patients are listed in Table 1. Healthy controls (male (m)/female (f): 13/8), paraplegics (m/f: 6/5), and tetraplegics (m/f: 11/1) did not significantly differ with respect to sex (p = 0.095). However, we found significant differences in age between groups (p = 0.002) with paraplegics being significantly older (58.1 ± 17.8 years) than controls (33.7 ± 9.8 years) (p = 0.001), while neither the difference between paraplegics and tetraplegics (41.1 ± 17.0 years) (p = 0.078) nor between tetraplegics and controls (p = 0.19) was significant. Tetra- and paraplegics did not significantly differ in their classification according to the AIS (p = 0.14). Among paraplegics, 2 were AIS A, 1 AIS B, 2 AIS C, and 6 AIS D; among tetraplegics, 5 were AIS A, 4 AIS B, 1 AIS C, and 2 AIS D. There were no significant differences in the time points of the MRI scans after injury between tetra- and paraplegics (baseline: p = 0.22, 2-months: p = 0.71, 6-months: p = 0.95, 1-year: p = 0.62, 2-years: p = 0.96).

### 3.2 Micro- and macrostructural changes of the corticospinal tracts and dorsal columns over 2 years

#### 3.2.1 Retrograde degenerative changes in the CST

**Fig. 2:**
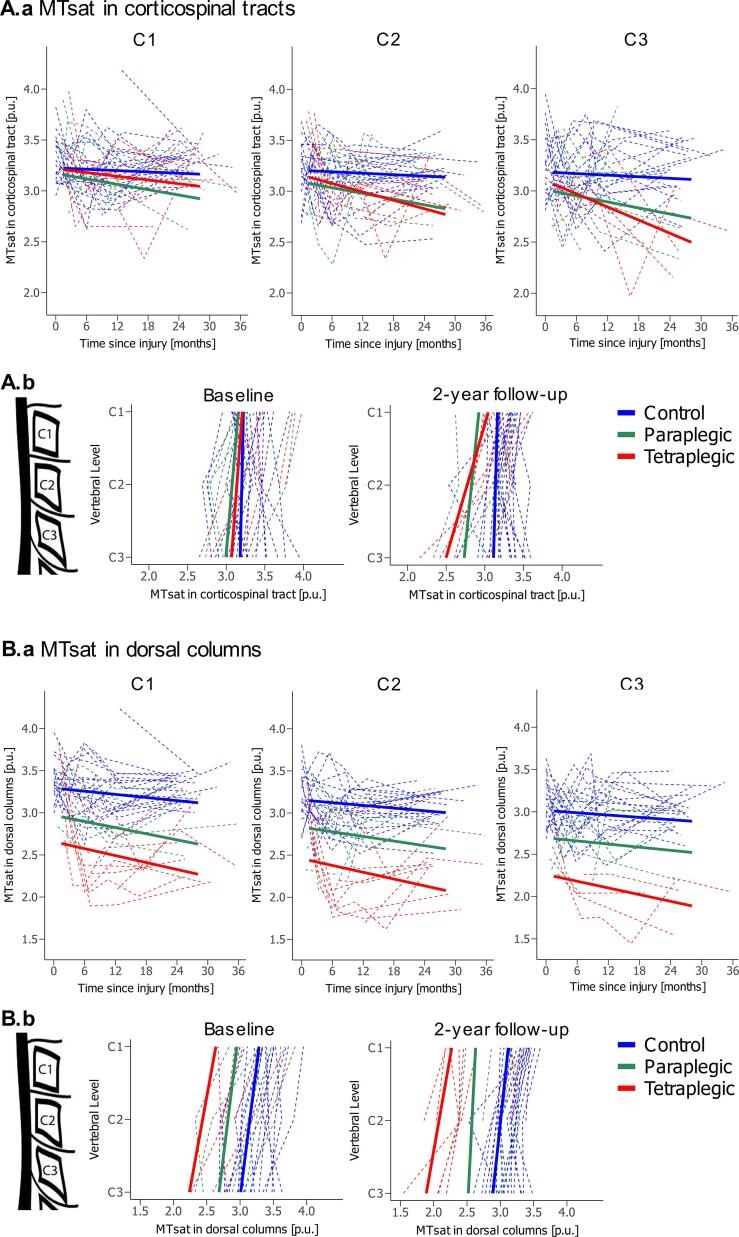
**Microstructural changes in the cervical cord over 2 years post-SCI.** (A.a) Change in MTsat (linear fit) within the CST of vertebral levels C1-C3 over 2 years post-injury and (A.b) change in MTsat within the CST across the cervical cord (C1-C3) at baseline and 2-years follow-up for healthy controls (blue), paraplegics (green), and tetraplegics (red). (B.a) Change in MTsat (linear fit) within the DC of vertebral levels C1-C3 over 2 years post-injury and (B.b) change in MTsat within the DC across the cervical cord (C1-C3) at baseline and 2-years follow-up for healthy controls (blue), paraplegics (green), and tetraplegics (red). *CST* corticospinal tracts, *DC* dorsal columns, *MTsat* magnetization transfer saturation. (For interpretation of the references to colour in this figure legend, the reader is referred to the web version of this article.)

Within the CST, MTsat was not significantly different between patients and controls at baseline (Table 4, Fig. 2A.a and 2A.b). Tetraplegics showed a greater linear decrease in MTsat over time compared to healthy controls over 2 years after injury at C2 (tetra vs controls: −0.011 p.u./month, p = 0.037, 95% CI −0.022 p.u./month to −0.001 p.u./month), while paraplegics were not significantly different to controls (Table 2, Fig. 2A.a). At 1-year follow-up, tetraplegics showed greater MTsat decreases within the CST at lower vertebral levels across C1-C3, resulting in a spatial gradient across the upper cervical cord when compared to healthy controls (tetra vs controls: −0.140 p.u./level, p = 0.015, 95% CI −0.257 p.u./level to −0.024 p.u./level). This spatial gradient increased further by 2-year follow-up (-0.247 p.u./level, p = 0.034, 95% CI −0.479 p.u./level to −0.016 p.u./level). Paraplegics did not develop this spatial gradient compared to controls over 2 years (Table 3, Table 4, Fig. 2A.b).

**Table 2:** Linear and quadratic rates of macro- and microstructural parameters at baseline.

	Rate at baseline per level			pSCI - controls			tSCI - controls			tSCI - pSCI		
Parameter	controls	pSCI	tSCI	difference	p-value	95% CI	difference	p-value	95% CI	difference	p-value	95% CI
MTsat CST [p.u./month]												
C1	−0.002	−0.009	−0.006	−0.007	0.55	−0.022 to 0.009	−0.004	0.76	−0.018 to 0.010	0.003	0.93	−0.015 to 0.020
C2	−0.002	−0.009	−0.014	−0.007	0.31	−0.018 to 0.004	−0.011	**0.037**	−0.022 to −0.001	−0.004	0.71	−0.018 to 0.009
C3	−0.003	−0.010	−0.021	−0.007	0.63	−0.027 to 0.012	−0.019	0.059	−0.038 to 0.001	−0.012	0.48	−0.036 to 0.013

MTsat DC [p.u./month]												
C1	−0.006	−0.012	−0.014	−0.006	0.69	−0.023 to 0.012	−0.008	0.45	−0.023 to 0.008	−0.002	0.98	−0.021 to 0.018
C2	−0.005	−0.009	−0.013	−0.004	0.76	−0.017 to 0.009	−0.008	0.21	−0.020 to 0.003	−0.004	0.76	−0.019 to 0.010
C3	−0.005	−0.006	−0.013	−0.002	0.97	−0.020 to 0.017	−0.009	0.49	−0.027 to 0.010	−0.007	0.75	−0.030 to 0.016

LRW [mm/month]												
C1	−0.001	−0.030	−0.018	−0.029	0.27	−0.074 to 0.015	−0.018	0.53	−0.056 to 0.021	0.012	0.84	−0.038 to 0.061
C2	0.001	−0.032	−0.035	−0.032	0.11	−0.070 to 0.006	−0.035	**0.037**	−0.069 to −0.002	−0.003	0.98	−0.045 to 0.039
C3	0.002	−0.034	−0.052	−0.035	0.18	−0.082 to 0.011	−0.053	**0.006**	−0.093 to −0.013	−0.018	0.70	−0.069 to 0.034

APW [mm/month]												
C1	0.006	−0.010	−0.053	−0.016	0.71	−0.066 to 0.033	−0.059	**0.006**	−0.104 to −0.015	−0.043	0.16	−0.097 to 0.012
C2	0.004	−0.016	−0.057	−0.019	0.57	−0.065 to 0.026	−0.061	**0.003**	−0.103 to −0.019	−0.041	0.13	−0.092 to 0.009
C3	0.001	−0.021	−0.061	−0.022	0.56	−0.073 to 0.029	−0.062	**0.005**	−0.108 to −0.016	−0.040	0.22	−0.097 to 0.017

	**Quadratic effect per level**			**pSCI - controls**			**tSCI – controls**			**tSCI - pSCI**		
Parameter	controls	pSCI	tSCI	difference	p-value	95% CI	difference	p-value	95% CI	difference	p-value	95% CI
LRW [mm/month^2^]												
C1	0.0002	0.0009	0.0001	0.0007	0.51	−0.0007 to 0.0021	−0.0001	0.97	−0.0013 to 0.0010	−0.0008	0.46	−0.0023 to 0.0008
C2	0.0002	0.0010	0.0004	0.0008	0.17	−0.0002 to 0.0018	0.0002	0.88	−0.0008 to 0.0011	−0.0006	0.43	−0.0018 to 0.0006
C3	0.0002	0.0011	0.0007	0.0009	0.30	−0.0005 to 0.0024	0.0005	0.60	−0.0007 to 0.0017	−0.0004	0.79	−0.0020 to 0.0012

APW [mm/month^2^]												
C1	−0.0002	0.0001	0.0010	0.0003	0.93	−0.0015 to 0.0021	0.0012	0.15	−0.0003 to 0.0028	0.0010	0.48	−0.0010 to 0.0029
C2	−0.0001	0.0002	0.0011	0.0004	0.83	−0.0012 to 0.0020	0.0012	0.14	−0.0003 to 0.0027	0.0008	0.53	−0.0010 to 0.0026
C3	0.0000	0.0004	0.0011	0.0005	0.79	−0.0013 to 0.0023	0.0011	0.21	−0.0005 to 0.0027	0.0006	0.72	−0.0014 to 0.0026

**Table 3:** Spatial gradient of macro- and microstructural parameters across cervical levels C1-C3 over 2 years.

	**Change per vertebral level**			**pSCI - controls**			**tSCI - controls**			**tSCI - pSCI**		
parameter	controls	pSCI	tSCI	difference	p-value	95% CI	difference	p-value	95% CI	difference	p-value	95% CI
**Baseline**												
MTsat CST [p.u.]	−0.020	−0.081	−0.070	−0.060	0.63	−0.220 to 0.100	−0.049	0.80	−0.236 to 0.137	0.011	0.99	−0.207 to 0.229
MTsat DC [p.u.]	−0.137	−0.134	−0.198	0.003	1.00	−0.144 to 0.151	−0.061	0.67	−0.236 to 0.113	−0.065	0.73	−0.270 to 0.141
LRW [mm]	0.257	0.149	0.193	−0.108	0.50	−0.338 to 0.122	−0.063	0.78	−0.290 to 0.164	0.045	0.91	−0.218 to 0.308
APW [mm]	−0.294	−0.316	−0.419	−0.023	0.96	−0.236 to 0.191	−0.125	0.33	−0.336 to 0.085	−0.102	0.57	−0.345 to 0.141

**2 months**												
MTsat CST [p.u.]	−0.021	−0.082	−0.086	−0.061	0.54	−0.201 to 0.080	−0.065	0.61	−0.231 to 0.102	−0.004	1.00	−0.197 to 0.189
MTsat DC [p.u.]	−0.135	−0.128	−0.198	0.008	0.99	−0.123 to 0.139	−0.062	0.61	−0.220 to 0.095	−0.070	0.63	−0.254 to 0.114
LRW [mm]	0.259	0.145	0.160	−0.114	0.45	−0.340 to 0.112	−0.099	0.53	−0.321 to 0.123	0.015	0.99	−0.242 to 0.271
APW [mm]	−0.299	−0.327	−0.427	−0.028	0.94	−0.239 to 0.183	−0.129	0.30	−0.336 to 0.079	−0.100	0.57	−0.339 to 0.138

**6 months**												
MTsat CST [p.u.]	−0.022	−0.084	−0.114	−0.062	0.38	−0.176 to 0.051	−0.093	0.24	−0.229 to 0.044	−0.031	0.89	−0.188 to 0.127
MTsat DC [p.u.]	−0.132	−0.116	−0.197	0.016	0.94	−0.095 to 0.127	−0.064	0.47	−0.198 to 0.069	−0.080	0.43	−0.235 to 0.075
LRW [mm]	0.262	0.141	0.106	−0.121	0.44	−0.359 to 0.117	−0.156	0.23	−0.384 to 0.072	−0.035	0.95	−0.301 to 0.231
APW [mm]	−0.306	−0.341	−0.442	−0.036	0.92	−0.256 to 0.185	−0.136	0.28	−0.348 to 0.076	−0.100	0.59	−0.347 to 0.146

**1 year**												
MTsat CST [p.u.]	−0.023	−0.087	−0.163	−0.064	0.35	−0.175 to 0.047	−0.140	**0.015**	−0.257 to −0.024	−0.076	0.40	−0.218 to 0.066
MTsat DC [p.u.]	−0.127	−0.097	−0.195	0.030	0.82	−0.090 to 0.149	−0.068	0.38	−0.192 to 0.055	−0.098	0.27	−0.249 to 0.054
LRW [mm]	0.268	0.143	0.033	−0.125	0.50	−0.391 to 0.141	−0.236	0.067	−0.484 to 0.013	−0.111	0.64	−0.407 to 0.185
APW [mm]	−0.313	−0.354	−0.465	−0.041	0.91	−0.284 to 0.201	−0.152	0.26	−0.380 to 0.077	−0.111	0.59	−0.381 to 0.160

**2 years**												
MTsat CST [p.u.]	−0.026	−0.095	−0.273	−0.069	0.78	−0.320 to 0.183	−0.247	**0.034**	−0.479 to −0.016	−0.179	0.32	−0.479 to 0.121
MTsat DC [p.u.]	−0.115	−0.054	−0.191	0.060	0.84	−0.197 to 0.318	−0.076	0.71	−0.317 to 0.165	−0.136	0.53	−0.447 to 0.174
LRW [mm]	0.278	0.184	−0.045	−0.094	0.77	−0.424 to 0.235	−0.323	**0.024**	−0.609 to −0.037	−0.229	0.29	−0.590 to 0.132
APW [mm]	−0.303	−0.324	−0.506	−0.022	0.98	−0.320 to 0.276	−0.203	0.16	−0.468 to 0.062	−0.181	0.38	−0.510 to 0.147

**Table 4:** Mean macro- and microstructural parameters at baseline and 2-year follow-up.

	**Mean per level**			**pSCI - controls**			**tSCI - controls**			**tSCI - pSCI**		
Parameter	controls	pSCI	tSCI	difference	p-value	95% CI	difference	p-value	95% CI	difference	p-value	95% CI
**Baseline**												
MTsat CST [p.u.]												
C1	3.27	3.20	3.25	−0.06	0.83	−0.33 to 0.21	−0.01	0.99	−0.29 to 0.26	0.05	0.93	−0.28 to 0.38
C2	3.25	3.12	3.18	−0.12	0.43	−0.37 to 0.12	−0.06	0.82	−0.32 to 0.19	0.06	0.87	−0.23 to 0.36
C3	3.23	3.04	3.11	−0.19	0.32	−0.49 to 0.12	−0.11	0.72	−0.47 to 0.24	0.07	0.90	−0.33 to 0.48

MTsat DC [p.u.]												
C1	3.36	3.02	2.71	−0.33	**0.015**	−0.61 to −0.06	−0.65	**<0.0001**	−0.93 to −0.36	−0.32	0.070	−0.65 to 0.02
C2	3.22	2.89	2.51	−0.33	**0.005**	−0.57 to −0.09	−0.71	**<0.0001**	−0.96 to −0.45	−0.38	**0.008**	−0.68 to −0.08
C3	3.08	2.76	2.31	−0.33	**0.024**	−0.61 to −0.04	−0.77	**<0.0001**	−1.10 to −0.44	−0.44	**0.019**	−0.83 to −0.06

LRW [mm]												
C1	10.81	11.03	10.79	0.22	0.57	−0.31 to 0.75	−0.01	1.00	−0.53 to 0.50	−0.23	0.61	−0.83 to 0.36
C2	11.06	11.18	10.99	0.11	0.88	−0.44 to 0.66	−0.08	0.93	−0.61 to 0.46	−0.19	0.74	−0.80 to 0.43
C3	11.32	11.32	11.18	0.00	1.00	−0.65 to 0.66	−0.14	0.85	−0.78 to 0.50	−0.14	0.88	−0.88 to 0.59

APW [mm]												
C1	7.95	7.88	7.67	−0.08	0.93	−0.58 to 0.43	−0.28	0.36	−0.78 to 0.21	−0.21	0.66	−0.77 to 0.36
C2	7.66	7.56	7.25	−0.10	0.87	−0.57 to 0.37	−0.41	0.094	−0.87 to 0.06	−0.31	0.35	−0.84 to 0.22
C3	7.37	7.24	6.83	−0.12	0.85	−0.66 to 0.41	−0.53	**0.046**	−1.06 to −0.01	−0.41	0.23	−1.01 to 0.19

**2 years**												
MTsat CST [p.u.]												
C1	3.21	2.97	3.09	−0.24	0.15	−0.55 to 0.07	−0.12	0.47	−0.37 to 0.13	0.12	0.64	−0.21 to 0.46
C2	3.18	2.87	2.82	−0.31	**0.014**	−0.56 to −0.06	−0.37	**0.0005**	−0.58 to −0.15	−0.06	0.87	−0.33 to 0.22
C3	3.16	2.78	2.54	−0.38	0.064	−0.78 to 0.02	−0.61	**0.0007**	−0.98 to −0.25	−0.24	0.45	−0.71 to 0.24

MTsat DC [p.u.]												
C1	3.19	2.70	2.34	−0.49	**0.022**	−0.92 to −0.06	−0.85	**<0.0001**	−1.20 to −0.49	−0.36	0.15	−0.82 to 0.10
C2	3.08	2.65	2.15	−0.43	**0.016**	−0.79 to −0.07	−0.92	**<0.0001**	−1.23 to −0.62	−0.49	**0.010**	−0.88 to −0.10
C3	2.96	2.59	1.96	−0.37	0.13	−0.82 to 0.09	−1.00	**<0.0001**	−1.42 to −0.58	−0.63	**0.017**	−1.16 to −0.10

LRW [mm]												
C1	10.95	10.86	10.38	−0.09	0.94	−0.79 to 0.60	−0.57	0.058	−1.15 to 0.02	−0.47	0.26	−1.20 to 0.25
C2	11.23	11.04	10.34	−0.19	0.81	−0.92 to 0.54	−0.89	**0.004**	−1.53 to −0.25	−0.70	0.082	−1.48 to 0.07
C3	11.51	11.23	10.29	−0.28	0.73	−1.18 to 0.62	−1.21	**0.002**	−2.02 to −0.41	−0.93	0.062	−1.90 to 0.04

APW [mm]												
C1	7.96	7.64	6.98	−0.32	0.52	−1.01 to 0.38	−0.98	**0.0009**	−1.59 to −0.37	−0.66	0.094	−1.41 to 0.09
C2	7.66	7.32	6.48	−0.34	0.35	−0.92 to 0.24	−1.18	**<0.0001**	−1.69 to −0.68	−0.84	**0.005**	−1.46 to −0.22
C3	7.36	6.99	5.97	−0.36	0.33	−0.97 to 0.25	−1.38	**<0.0001**	−1.91 to −0.86	−1.02	**0.001**	−1.67 to −0.37

**Fig. 3:**
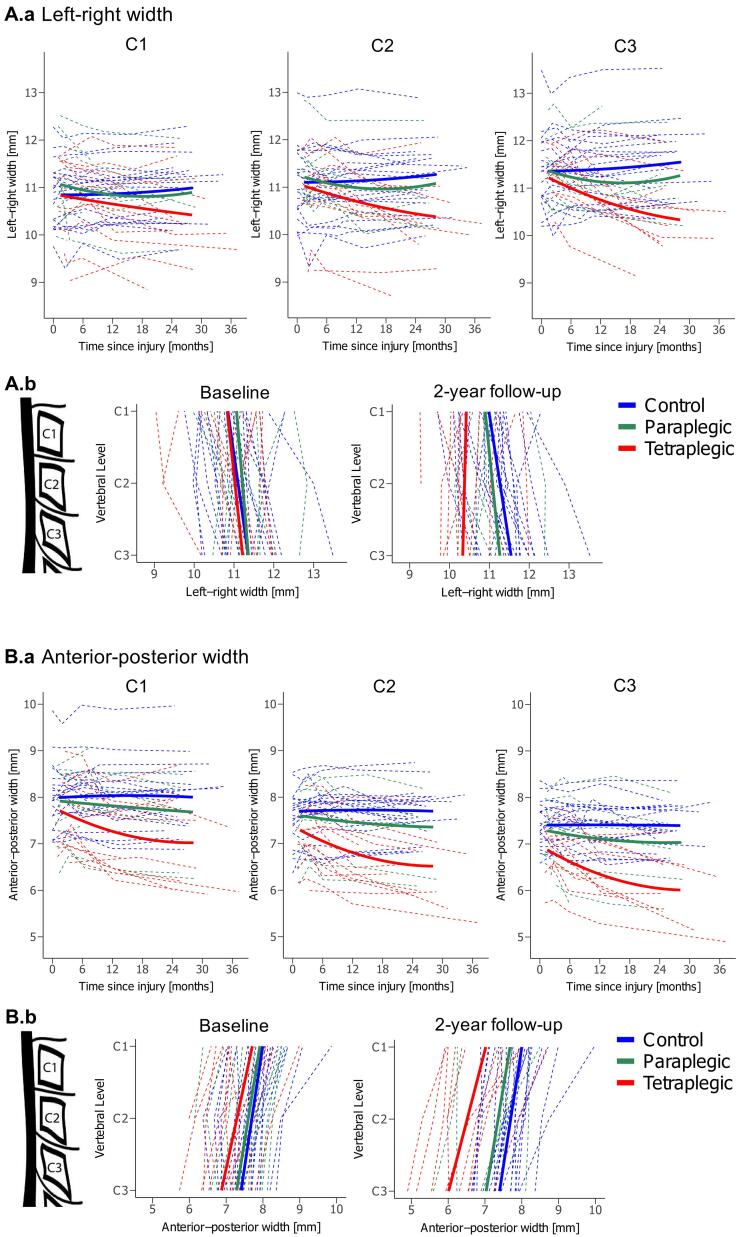
**Macrostructural changes in the cervical cord over 2 years post-SCI.** (A.a) Change in LRW (quadratic fit) of vertebral levels C1-C3 over 2 years post-injury and (A.b) change in LRW across the cervical cord (C1-C3) at baseline and 2-years follow-up for healthy controls (blue), paraplegics (green), and tetraplegics (red). (B.a) Change in APW (quadratic fit) of vertebral levels C1-C3 over 2 years post-injury and (B.b) change in APW across the cervical cord (C1-C3) at baseline and 2-years follow-up for healthy controls (blue), paraplegic (green), and tetraplegic (red). *APW* anterior-posterior width, *LRW* left–right width. (For interpretation of the references to colour in this figure legend, the reader is referred to the web version of this article.)

Similar to MTsat, there was no significant difference in LRW between patients and controls at baseline (Table 4, Fig. 3A.a and 3A.b). Linear decreases in LRW over time were greater in tetraplegics compared to healthy controls at C2 (tetra vs controls: −0.035 mm/month, p = 0.037, 95% CI −0.069 mm/month to −0.002 mm/month) and at C3 (-0.053 mm/month, p = 0.006, 95% CI −0.093 mm/month to −0.013 mm/month). Paraplegics did not differ significantly from controls. There was no difference in the deceleration of the LRW decrease between paraplegics and tetraplegics (Table 2, Fig. 3A.a). At 2-year follow-up, tetraplegics developed a spatial gradient across C1-C3 with greater decreases in LRW at lower vertebral levels (tetra vs controls: −0.323 mm/level, p = 0.024, 95% CI −0.609 mm/level to −0.037 mm/level). This difference between cervical levels was not observable in paraplegics (Table 3, Table 4, Fig. 3A.b).

#### 3.2.2 Anterograde degenerative changes in the DC

At baseline, MTsat within the DC was already lower in both tetraplegics and paraplegics compared to controls, where the differences were similar across C1-C3 (Table 4, Fig. 2B.a and 2B.b). The linear decrease in MTsat within the DC over time did not differ between controls and patients (Table 2, Fig. 2B.a). Consequently, both tetraplegics and paraplegics showed significantly lower MTsat within the DC at 2-year follow-up with similar magnitudes across C1-C3 (Table 3, Table 4, Fig. 2B.b).

In contrast to MTsat, APW did not differ significantly between SCI patients and controls at baseline (Table 4, Fig. 3B.a and 3B.b). However, the linear decrease in APW over time was greater in tetraplegics compared to controls at all three cervical levels with similar magnitudes (tetra vs controls; C1: −0.059 mm/month, p = 0.006, 95% CI −0.104 mm/month to −0.015 mm/month; C2: −0.061 mm/month, p = 0.003, 95% CI −0.103 mm/month to −0.019 mm/month; C3: −0.062 mm/month, p = 0.005, 95% CI −0.108 mm/month to −0.016 mm/month), while paraplegics were not significantly different to controls. The deceleration of the APW decrease was not significantly different between paraplegics and tetraplegics (Table 2, Fig. 3B.a). As the linear decrease in APW was similar at each vertebral level across C1-C3, no spatial gradient developed in tetraplegics compared to controls (Table 3, Table 4, Fig. 3B.b).

### 3.3 Association between clinical improvement and microstructural markers of myelination

**Fig. 4:**
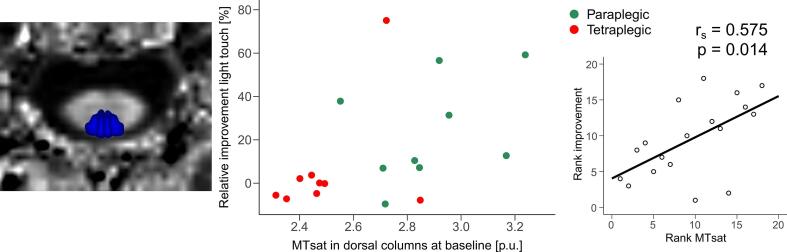
**Correlation between estimated relative improvement in light touch score and estimated microstructural parameter MTsat at baseline.** A higher relative improvement in light touch score was associated with higher MTsat within the dorsal columns at baseline (1.5 months post-injury) across cervical levels C1-C3. The plot on the right side illustrates the Spearman's rank correlation coefficient and the associated p-value between MTsat and the relative improvement in light touch score. *MTsat* magnetization transfer saturation.

Baseline MTsat values within the dorsal columns across cervical levels C1-C3 correlated with the relative improvement in the light touch score (r_s_ = 0.575, p = 0.014) (Fig. 4), while baseline MTsat values within the corticospinal tract were not associated with the relative improvement in the motor score. The temporal evolution of functional improvement (motor and light touch scores) is illustrated in Supplementary Figure S1.

## 4 Discussion

This study shows greater rates of retrograde neurodegeneration within the corticospinal tracts at levels closer to the lesion in the upper cervical cord following SCI. The differential rate of neurodegeneration across cervical levels leads to the gradual development of a spatial gradient in macro- and microstructural surrogates within the corticospinal tracts over two years after injury with more pronounced changes found closer to the lesion. In the dorsal columns – albeit significant reductions of micro- and macrostructural markers of neurodegeneration were evident – such a gradient was not detectable, with all cervical levels being similarly affected, suggesting that the magnitude of anterograde degeneration does not depend on the distance to the lesion. Importantly, our results support established histopathological findings (Becerra et al., 1995, Bresnahan, 1978, Daniel and Strich, 1969, Hill, 2017, Hill et al., 2001, Kalil and Schneider, 1975, Pallini et al., 1988, Seif et al., 2007) regarding the difference between the spatiotemporal dynamics of retrograde and anterograde neurodegeneration and demonstrate that advanced MRI sequences as non-invasive techniques are sensitive to detect early as well as late remote tract-specific neurodegenerative changes. Moreover, changes in myelin-sensitive MTsat in the dorsal columns were predictive of functional improvement, demonstrating the clinical relevance and predictive potential of these quantitative MRI readouts.

### 4.1 Anterograde and retrograde degeneration: Evidence from histology

Evidence from animal studies investigating anterograde and retrograde neurodegeneration following spinal cord injury suggests that these processes are fundamentally different in terms of their spatiotemporal development (Bresnahan, 1978, Daniel and Strich, 1969, Hill, 2017, Kalil and Schneider, 1975). Anterograde (Wallerian) degeneration, originally described by Augustus Waller in the peripheral nervous system (Waller and Owen, 1850), is a pathophysiological process that occurs in the spinal cord shortly after transection of nerve fibers. It proceeds through several stages including axonal degeneration, myelin sheath disintegration, which is followed by myelin protein and lipid breakdown and removal of myelin and axon debris (Becerra et al., 1995, Daniel and Strich, 1969). Wallerian degeneration is initiated very shortly after injury with morphological axonal changes occurring within the first 6 h after axotomy and myelin sheath breakdown products being found as early as 48 h after transection of the nerve fibers (Becerra et al., 1995, Beirowski et al., 2010, Daniel and Strich, 1969). The process was shown to continue one year after injury, which is mainly attributable to the protracted clearance of cell debris, and appears to be slower in the central than in the peripheral nervous system (Beirowski et al., 2010, Daniel and Strich, 1969). As the whole distal axonal segment is eventually affected by anterograde degeneration after injury to the proximal portion, pathologic changes can be detected at various vertebral levels (Becerra et al., 1995).

Retrograde degeneration refers to the gradual retraction and degeneration of the proximal end of the severed axon (Hill, 2017). It includes the formation of a retraction bulb at the transected axon stump and occurrence of degenerative myelin changes (Seif et al., 2007). Retraction bulbs, which can be detected as early as one day after injury, persist for several months (Pallini et al., 1988). In particular, axons within the CST were found to exhibit a prolonged dieback (Hill, 2017). Retrograde degeneration starts at the lesion site and progresses slowly towards the cell body, forming a neurodegenerative gradient (Ramon y Cajal, 1928).

### 4.2 A spatial gradient develops in tracts affected by retrograde but not by anterograde degeneration

Using advanced MRI sequences, our results showed a similar discrepancy in the spatiotemporal dynamics between retrograde and anterograde neurodegeneration. MTsat within the CST showed a greater decline over time in lower cervical levels giving rise to a spatial gradient with greater myelin changes in proximity to the lesion. This was supported by the macrostructural readout LRW, where, similar to MTsat within the CST, the linear decrease over time was greater at lower levels, gradually forming a spatial gradient over 2 years following SCI. In contrast, changes in MTsat within the DC over time were similar across cervical levels C1-C3. Likewise, cervical levels C1-C3 underwent significantly greater declines in APW with comparable magnitudes across levels. Thus, no spatial gradient across C1-C3 was evident in MTsat within the DC and in APW.

These results are consistent with the aforementioned histopathological findings, where retrograde degeneration was associated with a gradual axonal retraction and dieback from the lesion that explains the observed development of a spatial gradient in MTsat of the CST and LRW in tetraplegics (Hill, 2017, Hill et al., 2001, Kalil and Schneider, 1975, Pallini et al., 1988, Seif et al., 2007). In contrast, after transection or injury of the axonal fiber, the whole distal segment is affected by Wallerian degeneration due to the loss of the connection to the cell body (Becerra et al., 1995, Bresnahan, 1978, Daniel and Strich, 1969). This is in line with our observation that no significant spatial gradient occurred in MTsat of the DC and APW over 2 years. Therefore, we conclude that markers derived from advanced neuroimaging sequences are sensitive to changes observed in histological samples and offer a non-invasive alternative to track pathophysiological changes.

Moreover, we were able to unravel the cause of the spatial gradient across the cervical levels in terms of the temporal dynamics. Our results indicate that differences in the magnitude of atrophy rates (i.e. change/time) rather than differences in deceleration (i.e. the quadratic effect) underlie the formation of this gradient, as tetraplegics and paraplegics did not differ in the quadratic component. Hence, we did not find evidence that the distance to the lesion affects the deceleration of macrostructural neurodegeneration.

Furthermore, tetraplegics showed significantly greater changes in MTsat within the DC and APW when compared to paraplegics, which cannot simply be explained by the distance to the lesion given the absence of a spatial gradient in anterograde neurodegeneration. A factor that might contribute to this difference is the anatomical organization of the spinal cord. Axonal fibers enter respectively exit the spinal cord at each neurological level; thus, the number of fiber tracts within the spinal cord decreases at lower levels. Consequently, an injury at a high vertebral level (i.e. tetraplegics) could potentially affect more axons than an injury at a lower level (i.e. paraplegics), which could lead to a higher number of degenerating axons.

### 4.3 Microstructural readouts are more sensitive to early neurodegenerative changes

Both myelin-sensitive MTsat and APW detected neurodegenerative changes. However, there is a discrepancy between the temporal changes of MTsat within the DC and APW. While tetraplegics underwent a significant decrease in APW across all three cervical levels, MTsat did not change considerably over time. On the contrary, MTsat values were already decreased in both, paraplegics and tetraplegics compared to healthy controls at baseline with a greater magnitude in tetraplegics, suggesting that changes in myelination had occurred before the baseline measurement. This is in agreement with histopathological findings of remote Wallerian degeneration, which can be detected as early as eight days after injury (Becerra et al., 1995). APW on the other hand was not significantly different at the baseline measurement, indicating that cord macrostructure was not greatly affected by this time point, but rather showed signs of neurodegeneration with a temporal delay. This discrepancy in temporal evolution suggests higher sensitivity of microstructural measurements for detecting early neurodegenerative changes as compared to macrostructural readouts, as these macrostructural measures are influenced by several pathophysiological processes such as edema and cell infiltration, which might mask measurable atrophy (Ahuja et al., 2017). Higher sensitivity of quantitative MRI readouts has already been demonstrated in both SCI and degenerative cervical myelopathy patients and offers new possibilities for patient stratification and improving prognostication during the early stages after SCI (Martin et al., 2018, Seif et al., 2020, Severino et al., 2020).

### 4.4 Early microstructural changes are predictive of functional recovery

Functional recovery following SCI is limited and is typically observed during the first year after injury (Curt et al., 2008). However, some patients regain functionality beyond the first year while others show neurological worsening from the first to fifth year (Kirshblum et al., 2004). Crucial for the recovery of neurological deficits are axonal and synaptic plasticity which were previously found to occur both in the spinal cord and the brain with an early onset (days after injury) (Freund et al., 2013a, Ghosh et al., 2010, Sofroniew, 2018). Similarly, neurodegenerative processes affect both the brain and spinal cord and lead to measurable atrophy (Freund et al., 2013b, Grabher et al., 2015, Ziegler et al., 2018) and MRI signal changes (Fischer et al., 2021). These neurodegenerative processes were progressive and continued beyond two years following SCI (Ziegler et al., 2018). Further investigation is needed to disentangle the complex interplay between degenerative and regenerative mechanisms and how they relate to functional outcome. Nonetheless, we found evidence that quantitative markers of changes in myelination have clinical relevance and are predictive of functional improvement. In particular, baseline MTsat values within the DC correlated with the relative improvement in light touch score. Thus, quantitative MRI markers of tract-specific (de)myelination are not only sensitive to early pathophysiological changes on a microstructural level but are also indicative of the functional outcome after SCI. This opens new avenues for improving non-invasive prognostication especially during the early stages after injury by combining clinical examination and MRI. Moreover, the sensitivity of quantitative MRI to changes in microstructure is not exclusively restricted to SCI but can be generalized to improve prognostication in various demyelinating diseases such as multiple sclerosis (Granziera et al., 2021), degenerative cervical myelopathy (Paliwal et al., 2020), and amyotrophic lateral sclerosis (Barritt et al., 2018). In particular, the possibility of assessing tract-specific changes by quantitative MRI has the potential for improving function-specific prediction.

### 4.5 Limitations

This study has some limitations. Paraplegics were significantly older than both healthy controls and tetraplegics. However, we accounted for age as a covariate of no interest in all statistical analyses. Groups were heterogenous in terms of injury mechanism, neurological level of injury, and injury severity due to the exploratory nature of this study. Furthermore, the final sample size was relatively small (between 79% and 83% of original data for MPRAGE and between 64% and 74% for MTsat) due to data exclusion in case of susceptibility and/or motion artifacts; however, the compliance of subjects, a crucial factor for longitudinal study designs, was very high (missing data: 6%). During the study, the scanner was updated to a newer version which involved the change of certain hardware elements including the gradient coils. However, almost all subjects were scanned on both scanners, with the first time points being acquired on the older scanner, and later time points on the upgraded scanner. Since both groups (healthy controls and SCI patients) were recruited and scanned simultaneously, the scanner upgrade is not expected to affect group comparisons. Moreover, the statistical models were corrected for the scanner type in order to account for the scanner change. It should also be noted that although changes in MTsat were shown to be closely related to changes in myelination, MTsat is an indirect marker of myelin content and might also be affected by changes in other macromolecules (Georgiadis et al., 2021, Schmierer et al., 2004, Weiskopf et al., 2021). Similarly, LRW and APW serve as indirect markers for changes in the CST and DC (Grabher et al., 2015, Jutzeler et al., 2016, Lundell et al., 2011, Ziegler et al., 2018), as several other factors including changes in the gray matter and white matter pathways such as the spinothalamic and spinocerebellar tracts can also contribute to these measures. To differentiate between the effects of white matter and gray matter atrophy, future studies should consider acquiring sequences that allow the discrimination between GM and WM in the spinal cord, e.g. using T2*-weighted sequences. Moreover, macrostructural measures such as LRW and APW are crude markers of atrophy, as they do not provide information about the underlying pathophysiology. Finally, due to the FOV limited to the brain and the cervical cord we were not able to demonstrate whether a comparable spatial gradient exists in paraplegics in close proximity to the lesion as well, since the lesion site was below the investigated region.

## 5 Conclusion

In conclusion, this study shows that retrograde and anterograde degeneration exhibit different spatiotemporal dynamics following SCI. While anterograde degeneration was detectable from the earliest time point after injury, retrograde degeneration gradually developed and demonstrated a spatial neurodegenerative gradient. Tract-specific microstructural changes were predictive of functional improvement. Advanced MRI sequences can track these dynamic pathophysiological processes reliably and could potentially serve as biomarkers for regeneration and remyelination.

## Funding

This study was funded by the European Union's Horizon 2020 research and innovation program (No: 681094), the Swiss State Secretariat for Education, Research and Innovation (SERI) (No: 15.0137), the ERA-NET Neuron (No: 32NE30_173678), the Wings for Life Austria (WFL-CH-007/14), a SNF Eccellenza Professorial Fellowship grant (PCEFP3_181362/1), and a national MD-PhD scholarship by the SNF (323530_207038).

## CRediT authorship contribution statement

**Simon Schading:** Methodology, Formal analysis, Software, Visualization, Writing – original draft. **Gergely David:** Methodology, Formal analysis, Software, Writing – review & editing. **Tim Max Emmenegger:** Methodology, Software, Writing – review & editing. **Cristian Achim:** Formal analysis, Software, Writing – review & editing. **Alan Thompson:** Conceptualization, Writing – review & editing. **Nikolaus Weiskopf:** Conceptualization, Writing – review & editing. **Armin Curt:** Conceptualization, Writing – review & editing. **Patrick Freund:** Conceptualization, Methodology, Writing – review & editing, Resources, Supervision.

## Declaration of Competing Interest

The authors declare the following financial interests/personal relationships which may be considered as potential competing interests: SS reports no conflicts of interest. GD reports no conflicts of interest. TME reports no conflicts of interest. CA reports no conflicts of interest. AT reports personal fees paid to his institution from Eisai ltd and fees and support for travel from Hoffmann-La Roche outside the submitted work, and Editorial Board member, The Lancet Neurology, receiving free subscription, Editor-in-Chief, Multiple Sclerosis Journal, honorarium from SAGE Publications, support for travel as Chair, Scientific Advisory Committee, International Progressive MS Alliance, and member, National MS Society (USA), Research Programs Advisory Committee, Received honoraria and support for travel for lecturing from EXCEMED and Almirall. He acknowledges also support from the UCL/UCLH NIHR Biomedical Research Centre. NW reports that the Max Planck Institute for Human Cognitive and Brain Sciences has an institutional research agreement with Siemens Healthcare, that he holds a patent on acquisition of MRI data during spoiler gradients (US 10,401,453 B2) and that he was a speaker at an event organized by Siemens Healthcare and was reimbursed for the travel expenses. AC reports no conflicts of interest. PF reports no conflicts of interest.

## Data Availability

Data will be made available on request.
